# Risk for Premature Mortality and Intentional Self-harm in Autism Spectrum Disorders

**DOI:** 10.1007/s10803-020-04768-x

**Published:** 2020-11-02

**Authors:** Elina Jokiranta-Olkoniemi, David Gyllenberg, Dan Sucksdorff, Auli Suominen, Kim Kronström, Roshan Chudal, Andre Sourander

**Affiliations:** 1 Department of Child Psychiatry, University of Turku and Turku University Hospital, Lemminkäisenkatu 3/Teutori (3rd floor), 20014 Turku, Finland; 2 Department of Adolescent Psychiatry, University of Helsinki and Helsinki University Central Hospital, Helsinki, Finland; 3 National Institute of Health and Welfare, Helsinki, Finland; 4 Department of Adolescent Psychiatry, University of Turku and Turku University Hospital, Turku, Finland; 5 INVEST Research Flagship Center, University of Turku, Turku, Finland

**Keywords:** Autism, Mortality, Suicide, Psychiatric disorders, Natural cause

## Abstract

To examine the risk for premature mortality and intentional self-harm in autism spectrum disorders (ASD). Based on a national birth cohort. Children born in 1987–2005, diagnosed with ASD by 2007 (n = 4695) were matched with four non-ASD subjects (n = 18,450) and followed until 2015 for mortality and intentional self-harm. The risk among ASD subjects was elevated only for natural cause of death. The risk for intentional self-harm was increased in the unadjusted analyses, but decreased to non-significant after adjusting for comorbid psychiatric disorders. ASD subjects are at increased risk for premature mortality due to natural causes of death. While ASD subjects die of suicide and present with more self-harm, the association is explained by comorbid psychiatric disorders.

**Electronic supplementary material:**

The online version of this article (10.1007/s10803-020-04768-x) contains supplementary material, which is available to authorized users.

## Introduction

The increased risk for premature mortality among autism spectrum disorders (ASD) is well shown in clinical (Isager et al. 1999; Shavelle et al. 2001; Pickett et al. 2006; Mouridsen et al. 2008; Gillberg et al. 2010; Bilder et al. 2013) and population-based settings (Hirvikoski et al. 2016; Schendel et al. 2016). Reported risks for premature mortality among ASD subjects have varied from twofold (Pickett et al. 2006; Mouridsen et al. 2008; Gillberg et al. 2010; Hirvikoski et al. 2016; Schendel et al. 2016) to even tenfold (Bilder et al. 2013) compared with the non-ASD subjects. Previous findings suggest a relatively higher risk among female subjects (Isager et al. 1999; Shavelle et al. 2001; Pickett et al. 2006; Mouridsen et al. 2008; Gillberg et al. 2010; Hirvikoski et al. 2016; Schendel et al. 2016) and subjects with intellectual disability (ID) (Hirvikoski et al. 2016), but it remains unclear whether the risk is associated with other factors which may be modifiable such as comorbid psychiatric disorders.

Recent population-based studies suggest that a significant contributor for premature mortality in ASD is suicide (Hirvikoski et al. 2016; Schendel et al. 2016). However, suicidality in ASD is poorly understood and studies are scarce (Raja et al. 2011; Mayes et al. 2013; Cassidy et al. 2014), especially population-based studies (Hirvikoski et al. 2016; Schendel et al. 2016; Culpin et al. 2018). Both ASD and suicidality are relatively rare, and therefore population-based studies including the comparison with non-ASD subjects decrease the likelihood of selection bias and increase the generalizability of the findings. Population-based studies from Denmark (Schendel et al. 2016) and Sweden (Hirvikoski et al. 2016) reported between 5- to over sevenfold increased risk for intentional self-harm among ASD subjects compared with the non-ASD subjects. However, neither study was able to examine why this is the case. A large study published on suicide attempts in ASD reported that ASD itself is an independent risk factor even after adjusting for demographic data and psychiatric comorbidities (Chen et al. 2017). This finding, however, is in contrast with the findings from the population-based study showing that the risk of suicidality is associated with social communication impairments but not with ASD itself (Culpin et al. 2018).

In the present study we aimed to examine the risk of mortality and intentional self-harm among ASD subjects in a large Finnish national birth cohort including over a million births. The specific aims are to examine (1) mortality and causes of deaths and (2) intentional self-harm among ASD subjects compared to non-ASD subjects. The associations are examined adjusting for potential confounders such as comorbid psychiatric disorders, as well as psychiatric disorders in the family (parents and siblings). Given that previous findings suggested difference in the risk for mortality based on sex and comorbid ID, these examinations are conducted separately for males and females as well as among those with or without ID. We hypothesize that the ASD subjects are at increased risk for premature mortality and that their risk of intentional self-harm is explained by the comorbid psychiatric disorders.

## Methods

The present study is a part of The Finnish Prenatal Study of Autism Spectrum disorders (FIPS-A) register study, which has been described in detail previously (Lampi et al. 2011). The study is based on a national birth cohort including every live birth between the years 1987–2005 in Finland. Each child was followed up for the diagnosis of ASD to the end of 2007 and matched individually with four subjects without ASD, as described in more detail below. After the identification of subjects diagnosed with ASD and the non-ASD subjects, their family members (i.e. biological parents and siblings) were identified. To gather this data, a registry linkage between several nationwide Finnish registries was conducted by using personal identity code (for more details, see Lampi et al. 2011). Data were collected from four nationwide registries: the Finnish Hospital Discharge Register (FHDR), the Finnish Medical Birth Register (FMBR), the Finnish Central Population Register (FCPR) and the Finnish Cause of Death Register (FCDR) which are described in more detail below.

The FHDR includes nationwide computerized data from 1969 of all medical diagnoses, both somatic and psychiatric, made in public hospitals or inpatient wards of local health centres, military wards, and prison hospitals. Outpatient care in public specialized hospital units is included in the FHDR since 1998. The FHDR was used to identify subjects diagnosed with (1) ASD; (2) ID; (3) suicide attempt or other self-harm requiring inpatient or outpatient treatment; (4) psychiatric disorders among the subjects in the cohort and their family members.

The FMBR contains nationwide comprehensive data on all live births, stillbirths and the neonatal period up to the age of 7 days since 1987. The FMBR was used to identify non-ASD subjects and mothers and also to obtain the data on covariates.

The FCPR is a computerized nationwide register maintained by the Finnish population centre and local register offices. It contains basic information (e.g. name, address, municipality of residence, citizenship, family relations and deaths) of all permanent residents in Finland. The FCPR was used to obtain data of death (yes/no) and to identify the fathers and the siblings of the cohort members.

Causes of death were collected from the FCDR. The FCDR is maintained by Statistics Finland which has an archive of death certificates. The FCDR contains information on causes of death for Finnish citizens, who have died in Finland or abroad; and subjects who were domiciled in Finland at the time of death. The copies of death certificates can be obtained for research purposes as prescribed by law. Death certificates are completed by pathologists including additional information about cause and manner of death. The validity of the data in the FCDR has shown to be good (Lahti and Penttilä 2001).

The causes of death and psychiatric diagnoses are coded based on the International Classification of Diseases, Eighth Revision (ICD-8) (World Health Organization [WHO] 1967), from 1969 to 1986; International Classification of Diseases, Ninth Revision (ICD-9) (WHO 1977) from 1987 to 1995; and International Statistical Classification of Diseases and Related Health Problems, Tenth Revision (ICD-10) (WHO 1992) from 1996 onward.

The FIPS-A has received approval from the Ethical Committee of the Hospital District of Southwest Finland and from the institutional review board of the New York State Psychiatric Institute. The use of register data has been authorized by the Ministry of Social Affairs and Health in Finland and the National Institute for Health and Welfare.

### Study Population

Children born in Finland between 1987 and 2005, diagnosed with ASD (i.e. childhood autism [F84.0], Asperger’s syndrome [F84.5] or other pervasive developmental disorders/ pervasive developmental disorders—not otherwise specified [F84.8/F84.9]) at the end of 2007 were identified from the FHDR (n = 4695). They were individually matched 1:4 to subjects identified from the FMBR who were without the diagnosis of ASD or severe/profound ID, by sex, date of birth (within 1 month) and place of birth (n = 18,450). Subjects diagnosed with ID were identified from the FHDR by using the ICD-10 (F70–F79) and ICD-9 (317–319) codes. The parents and the full siblings of the cohort members were identified from the FMBR and the FCPR. Every member on the cohort was followed-up until December, 31st, 2015.

### Measures of Mortality and Intentional Self-harm

Each death certificate of the ASD subjects and non-ASD subjects was reviewed by 2 co-authors of the study (E.J.-O. and D.S.) and were categorized as follows: natural death (includes a variety of different causes such as cancer, congenital anomalies, pneumonia); accident; suicide; homicide or murder; unclassified. One non-ASD subject was deleted from the study because the lack of death certificate. Suicide was further categorized by the method as follows: shooting; hanging; intoxication; jumping from a high place; intentional traffic accident; drowning. This data, however, is not shown to protect the integrity of the individuals. Intentional self-harm was defined as suicide attempt or other intentional self-harm requiring inpatient treatment in public hospitals or outpatient treatment in public specialized hospital units. The data of intentional self-harm was gathered from the FHDR with ICD codes (ICD-8 codes: E950-E959; ICD-9 codes; E950-E959; ICD-10 codes: X60-X84, Y87.0, Z72.8, Z91.5).

### Covariates

Covariates chosen in to the analysis were maternal socioeconomic status (SES), comorbid psychiatric disorders, as well as psychiatric disorders, death and history of intentional self-harm among family member(s) (Croen et al. 2002; Jokiranta et al. 2013; Leyfer et al. 2006; O'Connor and Nock 2014; Turecki and Brent 2016). Data on maternal SES based on maternal occupation was obtained from the FMBR, categorized as follows: upper white collar; lower white collar; blue collar; others; missing. Comorbid psychiatric disorder(s) among subjects in the cohort as well as their family member(s) (biological parent(s) and/or sibling(s)) (yes/no) was gathered from the FHDR. Among ASD subjects and non-ASD subjects, comorbid psychiatric disorders were grouped as follows: non-affective psychoses; affective and anxiety disorders; substance-related disorders; disorders usually diagnosed in childhood (referred as childhood disorders); other disorders (see Appendix). Death among family member(s) was gathered from the FCPR. Intentional self-harm (excluding suicide) among the sibling and/or parent(s) (yes/no) was obtained from the FHDR and the FCDR. Data of covariates are presented in Table 1.

**Table 1:** Characteristics of the cohort and their association with (1) mortality among non-ASD subjects; (2) intentional self-harm among non-ASD subjects; and (3) ASD

	(1) Mortality among non-ASD subjects n = 18,450			(2) Intentional self-harm^a^ among non-ASD subjects n = 18,450			(3) ASD n = 23,146		
	No, n (%)	Yes, n (%)	P	No, n (%)	Yes, n (%)	P	ASD subject, n (%) n = 4696	Non-ASD subject, n (%) n = 18,450	P
Comorbid psychiatric disorder			.0003			< .0001			< .0001
Yes	3692 (20.1)	29 (38.2)		3696 (20.1)	25 (92.6)		3649 (77.7)	3721 (20.2)	
Non-affective psychoses	191 (1.0)	NS	.0021	189 (1.0)	6 (22.2)	< .0001	315 (6.7)	195 (1.1)	< .0001
Affective and anxiety disorders	1449 (7.9)	12 (15.8)	.0227	1443 (7.8)	18 (66.7)	< .0001	1126 (24.0)	1,461 (7.9)	< .0001
Substance-related disorders	510 (2.8)	10 (13.2)	< .0001	505 (2.7)	15 (55.6)	< .0001	158 (3.4)	520 (2.8)	.0442
Childhood disorders	2236 (12.2)	16 (21.1)	.0191	2243 (12.2)	9 (33.3)	.0013	3249 (69.2)	2252 (12.2)	< .0001
Other disorders	273 (1.5)	NS	.8761	274 (1.5)	0 (0.0)	NA	49 (1.0)	274 (1.5)	.0199
Psychiatric disorder among family member(s)			< .0001			.0125			< .0001
Yes	8260 (45.0)	57 (75.0)		8298 (45.0)	19 (70.4)		3064 (65.3)	8317 (45.1)	
Intentional self-harm^a^ among family member(s)			NA			NA			.0004
Yes	121 (0.7)	NS		123 (0.7)	0 (0.0)		55 (1.2)	123 (0.7)	
Death among family member(s)			.0264			NA			.0070
Yes	1316 (7.2)	11 (14.5)		1323 (7.2)	NS		392 (8.4)	1327 (7.2)	
Maternal socioeconomic status			< .0001			NA			.0045
Upper white collar workers	2272 (12.4)	7 (9.2)		2278 (12.4)	NS		559 (11.9)	2279 (12.4)	
Lower white collar workers	6367 (34.7)	15 (19.7)		6373 (34.6)	9 (33.3)		1523 (32.4)	6382 (34.6)	
Blue collar workers	2860 (15.6)	13 (17.1)		2872 (15.6)	NS		759 (16.2)	2873 (15.6)	
Others	2419 (13.2)	14 (18.4)		2427 (13.2)	6 (22.2)		696 (14.8)	2433 (13.2)	
Missing	4456 (24.3)	27 (35.5)		4473 (24.3)	10 (37.0)		1159 (24.7)	4483 (24.3)	

P-values calculated using the Poisson regression or logistic regression

*NS* not shown, if a cell includes 1 to 5 subjects; *NA* not applicable, i.e., could not be estimated due to small cell counts

*Excluding suicide

### Statistical Analysis

Prior to modelling the associations between ASD-status and mortality and intentional self-harm, bivariate analysis using the Poisson regression with p < .05 was conducted to assess the significance of the associations between the covariates and (1) mortality among non-ASD subjects; (2) intentional self-harm among non-ASD subjects and; (3) ASD. First we fitted unadjusted models, followed by models adjusted for covariates. Additional analysis investigated intentional self-harm more thoroughly by adjusting the model with different psychiatric disorder categories separately (i.e., non-affective psychoses; affective and anxiety disorders; substance-related disorders; childhood disorders; other disorders). To take into account the intracluster correlation in matched design, we used the Cox marginal hazards model with a robust sandwich covariance matrix to associate predictors, i.e., ASD and ID, with hazards of mortality, cause of death and intentional self-harm. The modelling was first conducted including all subjects with interaction for ASD and sex, and then stratified by ID of the ASD subjects. Effect modification was conducted to investigate, whether psychiatric comorbidities increased the risk of self-harm to the same degree among ASD subjects and non-ASD subjects. Associations were determined by using hazard ratios (HRs) in the marginal model with 95% confidence intervals (CIs). Statistical significance was judged with P-values calculated using Wald’s χ^2^-test or using 95% CIs. The level of significance was determined as p < .05. Due to the sensitive nature of the data, cells including 1 to 5 subjects are not shown. The statistical analyses were carried out using SAS 9.4 (SAS Institute Inc. Cary, NC, 2010).

## Results

The study consisted of 4695 ASD subjects and 18,450 non-ASD subjects. The mean age of ASD diagnosis was 8.0 years (SD 3.8 years). The mean age at the end of follow-up among ASD subjects was 21.5 years (SD 3.9 years) and among non-ASD subjects 21.5 years (SD 3.8 years). Among the ASD subjects, 3737 were boys, and 596 had ID. All covariates, except for intentional self-harm among family member(s), tested were associated with both mortality among the non-ASD subjects and ASD subject status (Table 1) and were therefore included in the adjusted analyses examining the risk for mortality in ASD. Intentional self-harm was only associated with two covariates; comorbid psychiatric disorders and psychiatric disorder among family member(s) (Table 1), and therefore these covariates were included in the adjusted analyses examining the risk for intentional self-harm in ASD.

### Premature Mortality

**Fig. 1:**
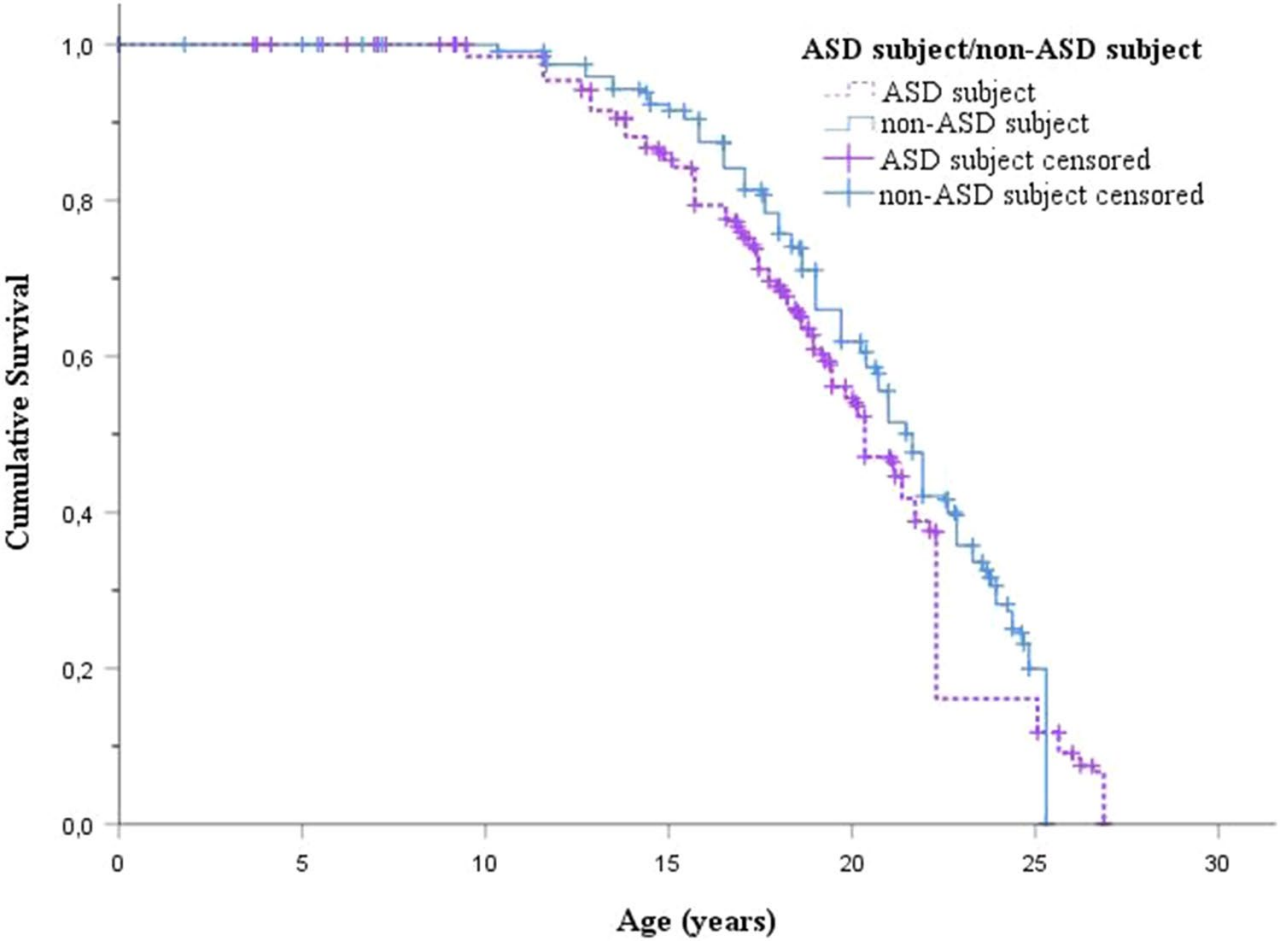
Age of premature mortality or end of the follow-up among ASD and non-ASD subjects

A survival curve (Fig. 1) shows the age of premature mortality among ASD and non-ASD subjects, as well as the impact of censoring due to end of follow-up. The mean age of death was 18.1 years (SD 5.8 years) among ASD subjects and 16.8 years (SD 5.5 years) among non-ASD subjects. Altogether 53 ASD subjects (1.1%) and 76 non-ASD subjects (0.4%) had died, majority were boys (79% among ASD subjects vs. 93% among non-ASD subjects) (Table 2). Majority of the ASD females who died, died due to natural cause (82%). Suicide was further categorized by the method (shooting; hanging; intoxication; jumping from a high place; intentional traffic accident; drowning) and there were 1 to 4 subjects in each group (data not shown). Both ASD subjects and non-ASD subjects used violent (shooting, hanging) and non-violent suicide methods.

**Table 2:** The number with premature death among ASD subjects and non-ASD subjects, stratified by intellectual disability

	Total		Male		Female		No intellectual disability among ASD subject		Intellectual disability among ASD subject	
	ASD subject n = 4695	Non-ASD subject n = 18,450	ASD subject n = 3737	Non-ASD subject n = 14,685	ASD subject n = 959	Non-ASD subject n = 3765	ASD subject n = 4100	Non-ASD subject n = 16,106	ASD subject n = 596	Non-ASD subject n = 2344
Mortality	53 (1.1%)	76 (0.4%)	42 (1.1%)	71 (0.5%)	11 (1.1%)	5 (0.1%)	35 (0.9%)	70 (0.4%)	18 (3.0%)	6 (0.3%)
Cause of death^a^										
Natural	27 (50.9%)	15 (19.7%)	18 (42.9%)	14 (19.7%)	9 (81.8%)	NS	12 (34.3%)	14 (20.0%)	15 (83.3%)	NS
Accident	13 (24.5%)	29 (38.2%)	12 (28.6%)	27 (38.0%)	NS	NS	10 (28.6%)	29 (41.4%)	NS	0 (0.0%)
Homicide or murder	0 (0.0%)	6 (7.9%)	0 (0.0%)	6 (8.5%)	0 (0.0%)	0 (0.0%)	0 (0.0%)	6 (8.6%)	0 (0.0%)	0 (0.0%)
Suicide	12 (22.6%)	23 (30.3%)	11 (26.2%)	21 (29.6%)	NS	NS	12 (34.3%)	19 (27.1%)	0 (0.0%)	NS

*NS* not shown, if a cell includes 1 to 5 subjects

^a^The numbers in category ‘unclear’ are not shown due to < 5 subjects in each cell

Table 3 shows the risk for premature mortality among ASD subjects and non-ASD subjects, stratified by ID. As shown in the table, in unadjusted analysis the risk for mortality among ASD subjects was nearly threefold (p =  < .0001), but after adjusting for covariates the risk decreased to nearly twofold (p = .0085). Among ASD females the risk for mortality was nearly ninefold (p =  < .0001) as compared with the controls in unadjusted analysis and the significant association remained in adjusted analysis showing over fivefold increased risk for premature mortality (p = .0040). Among ASD males the risk for mortality was over twofold (p =  < .0001) as compared with the controls in unadjusted analysis. After adjusting for covariates, however, the risk became non-significant (p = .0650). Based on interaction analysis, the relative increase in hazard of mortality associated with ASD was greater for females than for males (p = .0215). The risk for premature mortality was elevated both among ASD subjects with ID (HR = 12.1, 95% CI 4.4–32.9, p =  < .0001) and without ID (HR = 2.0, 95% CI 1.3–2.9, p = .0008) in unadjusted analysis, but after adjusting for covariates, the risk was significantly (p =  < .0001) elevated only among ASD subjects with ID showing over 11-fold risk for mortality. Cause of death examination showed in unadjusted analysis that ASD subjects are at increased risk of death due to natural cause (HR = 7.1, 95% CI 3.8–13.4, p =  < .0001) and suicide (HR = 2.1, 95% CI 1.02–4.1, p = .0431), as compared with the controls. In adjusted analysis, however, the risk was only observed in natural cause of death (HR = 6.1, 95% CI 2.7–13.5, p =  < .0001) except among ASD subjects without ID (HR = 2.5, 95% CI 0.9–6.8, p = .0677).

**Table 3:** The risk for premature death among ASD subjects and non-ASD subjects, stratified by intellectual disability

	Total		Male		Female		No intellectual disability among ASD subject		Intellectual disability among ASD subject	
	ASD subject n = 4696	Non-ASD subject n = 18,450	ASD subject n = 3737	Non-ASD subject n = 14,685	ASD subject n = 958	Non-ASD subject n = 3765	ASD subject n = 4099	Non-ASD subject n = 16,106	ASD subject n = 596	Non-ASD subject n = 2344
Mortality										
Unadjusted HR (95% CI)	2.8 (2.0–3.9) p = < .0001		**2.3 (1.6–3.4)** p = < .0001		**8.7 (3.0–25.1)** p = < .0001		2.0 (1.3–2.9) p = .0008		12.1 (4.4–32.9) p = < .0001	
Adjusted HR (95% CI)	1.7 (1.2–2.6) p = .0085		**1.5 (0.98–2.3)** p = .0650		**5.3 (1.7–16.3)** p = .0040		1.1 (0.7–1.8) p = .5637		11.5 (4.0–32.9) p = < .0001	
Cause of death										
Natural										
Unadjusted HR (95% CI)	7.1 (3.8–13.4) p = < .0001		5.1 (2.5–10.2) p = .0007		35.6 (4.5–281.7) p = < .0001		3.4 (1.6–7.3) p = .0020		60.3 (7.9–458.8) p = < .0001	
Adjusted HR (95% CI)	6.1 (2.7–13.5) p = < .0001		4.4 (2.0–9.8) p = .0003		29.6 (3.2–275.1) p = .0029		2.5 (0.9–6.8) p = .0677		67.4 (8.8–516.5) p = < .0001	
Accident										
Unadjusted HR (95% CI)	1.8 (0.9–3.3) p = .0812		1.8 (0.9–3.4) p = .0980		2.0 (0.2–21.8) p = .5778		1.4 (0.7–2.8) p = .3933		NA	
Adjusted HR (95% CI)	1.5 (0.7–3.0) p = .2811		1.5 (0.7–3.00) p = .3004		1.6 (0.1–18.12) p = .7100		1.2 (0.5–2.7) p = .6671		NA	
Unclear										
Unadjusted HR (95% CI)	1.3 (0.1–12.6) p = .8134		1.3 (0.1–12.6) p = .8134		1.0 (0.98–1.0) p = .9989		2.0 (0.2–21.7) p = .5808		NA	
Adjusted HR (95% CI)	NA		NA		NA		0.9 (0.07 -12.7) p = .9676		NA	
Suicide										
Unadjusted HR (95% CI)	2.1 (1.02–4.1) p = .0431		2.1 (0.99–4.3) p = .0518		2.0 (0.2–21.8) p = .5783		2.5 (1.2–5.1) p = .0136		NA	
Adjusted HR (95% CI)	0.8 (0.4–1.6) p = .5297		0.8 (0.4–1.7) p = .5512		0.7 (0.06–8.7) p = .8121		0.9 (0.4–1.8) p = .7034		NA	

Adjusted for maternal SES; comorbid psychiatric disorder(s); psychiatric disorders among family member(s); death in the family

*ASD* Autism spectrum disorders; *CI* confidence interval; *HR* hazard ratio; *NA* not applicable, i.e., HRs could not be estimated due to small cell counts; statistically significant interaction bolded

### Intentional Self-harm

The mean age of intentional self-harm was 18.7 years (SD 4.3 years) among ASD subjects and 19.7 years (SD 3.7 years) among non-ASD subjects. In total, 18 ASD subjects (0.4%) and 27 non-ASD subjects (0.2%) had been treated due to intentional self-harm (Table 4). Of those, majority were boys (11 ASD subjects [61%] vs. 18 non-ASD subjects [67%]). Table 4 shows the risk for intentional self-harm among ASD subjects and non-ASD subjects. In unadjusted analysis, the risk for intentional self-harm among ASD subjects was over 2.5-fold (p = .0015). However, after adjusting for comorbid psychiatric disorders, the magnitude of the association decreased to 0.8 (95% CI 0.4–1.5, p = .4673). Further adjustment with different psychiatric disorders categories showed that the associations became non-significant in each disorder category examined except for substance-related (HR 2.6, 1.5–4.8, p = .0014) and other disorders (HR 2.6, 95% CI 1.4–4.8, p = .0016). Also after further adjustment with both comorbid psychiatric disorders as well as psychiatric disorders among family members, the magnitude of the association remained 0.8 (95% CI 0.4–1.5, p = .4163) (Table 4). The same pattern of change from significant to non-significant association was also observed among ASD males, ASD females and among ASD subjects without ID, after adjustments with psychiatric comorbidity, with different psychiatric disorders categories and with both comorbid psychiatric disorders as well as psychiatric disorders among family member (Table 4). Among subjects with ID, the association was not significant in any phase. The investigation of effect modification showed that psychiatric comorbidities increased the risk of self-harm among ASD and non-ASD subjects in all categories examined except in “childhood disorders”, where the increased risk of self-harm was only observed among non-ASD subjects (HR = 3.7, 95% CI 1.7–8.3, p = .0013), but not among ASD subjects (HR = 2.5, 95% CI 0.7–8.6, p = .1493) (data not shown). An additional analysis examined the risk for intentional self-harm by combining suicide and suicide attempt or other intentional self-harm into one variable. The results remained the same, i.e., after adjusting with comorbid psychiatric disorders the associations became non-significant (p = .7776).

**Table 4:** The risk for intentional self-harm among ASD subjects and non-ASD subjects, stratified by intellectual disability

	Total			Male		Female		No intellectual disability among ASD subject		Intellectual disability among ASD subject	
	ASD subject n = 4695	Non-ASD subject n = 18,450		ASD subject n = 3737	Non-ASD subject n = 14,685	ASD subject n = 958	Non-ASD subject n = 3765	ASD subject n = 4099	Non-ASD subject n = 16,106	ASD subject n = 596	Non-ASD subject n = 2344
Intentional self-harm	18 (0.4%)		27 (0.2%)	11 (0.3%)	18 (0.1%)	7 (0.7%)	9 (0.2%)	17 (0.4%)	24 (0.2%)	NS	NS
Unadjusted HR (95% CI)	2.7 (1.5–5.1) p = .0015			2.4 (1.1–5.1) p = .0216		3.1 (1.1–8.3) p = .0255		2.8 (1.5–5.2) p = .0012		1.3 (0.1–12.8) p = .8050	
Adjusted^1^ HR (95% CI)	0.8 (0.4–1.5) p = .4673			0.7 (0.3–1.5) p = .4087		0.9 (0.3–2.5) p = .9124		0.8 (0.4–1.6) p = .5985		0.4 (0.04–3.9) p = .4334	
Adjusted with non-affective psychoses	1.6 (0.8–3.1) p = .2062			1.5 (0.7–3.5) p = .2879		1.6 (0.5–4.5) p = .4087		1.7 (0.8–3.5) p = .1691		0.7 (0.1–5.8) p = .7837	
Adjusted with affective and anxiety disorders	1.3 (0.7–2.5) p = .3435			1.2 (0.6–2.5) p = .6913		1.7 (0.6–4.7) p = .2907		1.3 (0.7–2.5) p = .4115		1.2 (0.1–11.0) p = .8591	
Adjusted with substance-related disorders	2.6 (1.5–4.8) p = .0014			2.4 (1.1–5.0) p = .0245		3.2 (1.2–8.7) p = .2091		2.6 (1.2–4.8) p = .0028		6.9 (0.5–87.6) p = .1380	
Adjusted with childhood disorders	1.3 (0.6–2.7) p = .5654			1.2 (0.5–2.9) p = .7130		1.4 (0.5–4.3) p = .5551		1.4 (0.6–3.3) p = .3760		0.5 (0.05–4.6) p = .5066	
Adjusted with other disorders	2.6 (1.4–4.8) p = .0016			2.4 (1.1–5.1) p = .0214		3.0 (1.1–8.2) p = .0281		2.8 (1.5–5.1) p = .0014		1.4 (0.1–13.3) p = .7714	
Adjusted^2^ HR (95% CI)	0.8 (0.4–1.5) p = .4163			0.7 (0.3–1.5) p = .3856		0.9 (0.3–2.4) p = .8415		0.8 (0.4–1.6) p = .5436		0.4 (0.04–3.8) p = .4205	

Adjusted^1^ for comorbid psychiatric disorder(s)

Adjusted^2^ for comorbid psychiatric disorder(s) and psychiatric disorders among family members

*ASD * Autism spectrum disorders; *CI*   confidence interval; *HR*   hazard ratio; *NS * not shown, if a cell includes 1 to 5 subjects

## Discussion

This study examined the risk for premature mortality and intentional self-harm among ASD subjects and non-ASD subjects by using a large national birth cohort and adjusting for several covariates. There are two main findings. First, the risk for premature mortality was increased among ASD subjects only for deaths due to natural causes. Second, the risk for intentional self-harm among ASD subjects was explained by the comorbid psychiatric disorders.

The risk for premature mortality was nearly twofold among ASD subjects as compared with the non-ASD subjects. This result is in line with the existing literature (Pickett et al. 2006; Mouridsen et al. 2008; Gillberg et al. 2010; Hirvikoski et al. 2016; Schendel et al. 2016). The risk for premature mortality was increased among ASD females and subjects with ID as compared with the non-ASD subjects, which is also in accordance with the previous studies (Isager et al. 1999; Shavelle et al. 2001; Pickett et al. 2006; Mouridsen et al. 2008; Gillberg et al. 2010; Bilder et al. 2013; Schendel et al. 2016). In addition, given that in the present study the majority of ASD subjects were males, we showed that the risk for mortality was relatively greater among ASD females as compared with the ASD males. However, contrary to other population-based studies (Hirvikoski et al. 2016; Schendel et al. 2016), ASD males and ASD subjects without ID did not have an increased risk for premature mortality. It should be noted that in unadjusted analysis, ASD males and ASD subjects without ID showed an increased risk for premature mortality, but after adjusting for several covariates (including maternal SES, comorbid psychiatric disorders, as well as psychiatric disorders and death in the family), the observed associations diminished. As previous population-based studies did not adjust for any (Hirvikoski et al. 2016) or only one (Schendel et al. 2016) of the covariates in this study (i.e., parental psychiatric disorders), our results suggest that the risk might be explained by several factors. Cause of death examination showed that ASD subjects had over sixfold increased risk for dying due to natural causes as compared with non-ASD subjects. In adjusted analysis, the increased risk was not seen for other causes examined. Our results showed that the risk for dying due to natural causes was especially high among ASD females and subjects with ID. These findings are also in line with the existing literature (Woolfenden et al. 2012) and expected given that ASD females are at higher risk for ID and medical conditions such as epilepsy (Amiet et al. 2008; Jokiranta et al. 2014).

Also as reported in the previous population-based studies (Hirvikoski et al. 2016; Schendel et al. 2016), in the present study the risk was elevated for suicide. However, although significant in the unadjusted analysis, when factors related to the family as well as comorbid psychiatric conditions are taken into account, the association was not significant. We also analyzed intentional self-harm including suicide attempts and other self-harm treated in the hospitals and clinics. In this analysis, we observed that even though ASD subjects present more intentional self-harm than the non-ASD subjects, the association is explained by comorbid psychiatric disorders. An additional analysis showed that the risk was specifically related with non-affective psychoses, affective and anxiety disorders as well as with childhood disorders. We also examined the risk for intentional self-harm by combining suicide and suicide attempt and other intentional self-harm into one variable, even though we are aware that mixed outcome measures (e.g. combination of suicide attempts and suicide) are not recommended (Segers and Rawana 2014). Also in this analysis we observed the same result, i.e., the association was explained by comorbid psychiatric disorders. This finding, however, is in contrast to the recently published study from Taiwan (Chen et al. 2017) showing that ASD itself is an independent risk factor for suicide attempts even after adjusting for psychiatric comorbidities. Opposing results might be due to different definitions of psychiatric disorders, because our category included a wider spectrum of different disorders. Our finding is of importance, as comorbid psychiatric conditions are common (Leyfer et al. 2006) and often modifiable risk factors among ASD subjects. However, the core symptoms of ASD including impairments in social interactions and communication may reduce the ability to seek help and treatment for psychiatric disorders, as previously suggested (Hirvikoski et al. 2016). Thus, clinicians working with the ASD subjects should pay particular attention to offering effective treatment for psychiatric disorders among ASD subjects.

The strengths of this study include a large nationwide register-based sample of ASD subjects and non-ASD subjects together with several covariates, enabling an in-depth analysis of mortality and intentional self-harm. The findings should be considered in the light of following limitations. First, the ASD subjects were identified from the FHDR which covers subjects identified from specialized health services. Individuals with less severe symptoms may not seek for care and therefore will not be found in the register. However, considering the availability in Finland of free universal health care for children and around fifteen health checkups of children by the age of 5 years, we expect the number of missing subjects to be relatively small. Second, this study examined children, adolescents and young adults (i.e., born in 1987–2005 and followed-up until the end of 2015) and therefore the results cannot be extrapolated beyond the age at follow-up. A longer follow-up later into adulthood could provide a more clear understanding of the mortality pattern. Third, given that this study was based on register data instead of personal contact and interviews, factors potentially related to self-harm (e.g. suicide ideation or plans) or the severity of the treated self-harm cannot be estimated. However, as we observed only diagnoses given either in inpatient treatment in public hospitals or in outpatient treatment in public specialized hospital units (not in primary health care), it is likely that these diagnoses reflect the severity of self-harm. Fourth, some of the diagnoses may be misclassified. On the other hand, in Finland children suspected to have ASD are referred from primary health care to more specialized services to be fully assessed by a multiprofessional, specialized team including child neurologists or psychiatrists, psychologists, and speech therapists using standardized methods. Thus, the FHDR has shown good validity for ASD (Lampi et al. 2010), but also for other disorders (Leivonen et al. 2014; Joelsson et al. 2016). Fifth, some of the analyses were based on small cell counts and therefore HRs could not be estimated. Hence, further studies are needed to explore this topic in more detail.

To conclude, this study confirms the previous findings reporting that ASD subjects are at increased risk for premature mortality. We showed that the increased risk was due to natural causes of death, but not for other causes examined. We also showed that even though ASD subjects die of suicide and present with more self-harm than do the non-ASD subjects, the association is explained by comorbid psychiatric disorders. Our findings warrant efforts to efficiently recognize and treat comorbid conditions among ASD subjects.

## Electronic supplementary material

Below is the link to the electronic supplementary material.

Supplementary file1 (DOCX 15 kb)
