# Supplementary material for: Risk for Premature Mortality and Intentional Self-harm in Autism Spectrum Disorders
Source: J Autism Dev Disord. 2020 Nov 2;51(9):3098–108. doi: 10.1007/s10803-020-04768-x (PMC8349316; doi:10.1007/s10803-020-04768-x)
Supplement: Supplementary file 1 — Supplementary file1 (DOCX 15 kb) [file 10803_2020_4768_MOESM1_ESM.docx]

| **Appendix.** **Diagnostic classification of comorbid psychiatric disorders according to the ICD-10 and corresponding ICD-9 diagnoses.** | | |
| --- | --- | --- |
| **Diagnostic group** | **Current classification ICD-10**  **(1996 to present)** | **ICD-9**  **(1987-1995)** |
| **Any comorbid psychiatric disorder** | F10-F99, excluding F84 and F70-F79 | 291-319, excluding 316, 299 and 317-319 |
| **Non-affective psychoses** | F20 schizophrenia, F21 schizotypal disorder, F22 delusional disorder, F23 acute polymorphic psychotic disorder without symptoms of schizophrenia, F24 induced delusional disorder, F25 schizoaffective disorder, F28 other nonorganic psychotic disorders, F29 unspecified nonorganic disorders | 295, 297, 2989X, 3012C |
| **Affective and anxiety disorders** | F30 hypomania, F31 bipolar affective disorder, F32 depressive episode, F33 recurrent depressive episode, F34 cyclothymia and dysthymia, F38 other single mood (affective) disorder, F39 unspecified mood (affective) disorder, F40 phobic anxiety disorders, F41 other anxiety disorders, F42 obsessive– compulsive disorder | 296, 3004A, 2988A, 3000A, 3000B, 3000C, 3002B, 3002C, 3002D, 3002X, 3003A |
| **Substance-related disorders** | Mental and behavioural disorders due to use of… F10 alcohol, F11 opioids, F12 cannabinoids, F13 sedatives or hypnotics, F14 cocaine, F15 other stimulants, including caffeine, F16 hallucinogens, F17 tobacco, F18 volatile solvents, F19 multiple drug use and use of other psychoactive substances | 303-305, 291-292 |
| **Disorder usually diagnosed in childhood (referred as childhood disorders)** | F80 specific developmental disorders of speech and language, F81 Specific developmental disorders of scholastic skills, F82 specific developmental disorder of motor function, F83 mixed specific developmental disorders, F88 other disorders of psychological development, F89 unspecified disorder of psychological development, F90 hyperkinetic disorders, F91 conduct disorders, F92 mixed disorders of conduct and emotions, F93 emotional disorders with onset specific to childhood, F94 disorders of social functioning with onset specific to childhood and adolescence, F95 tic disorders, F98 other behavioural and emotional disorders with onset usually occurring in childhood and adolescence, F51.3 sleepwalking, F51.4 sleep terrors | 313, 314, 315, 3120A, 3123C, 3123D, 3070A, 3070B, 3072A, 3072B, 3072C, 3072D, 3073A, 3074G, 3075D, 3076A, 3076B, 3076C, 3077A, 3092A, 3092B |
| **Other psychiatric disorder** | F10-F99, excluding F84, F70-F79 and the disorders in the above mentioned categories | 291-319, excluding 316, 299, 317-319 and the disorders in the above mentioned categories |
